# Sex-Related Differences in Medically Treated Moderate Aortic Stenosis

**DOI:** 10.1016/j.shj.2022.100042

**Published:** 2022-06-08

**Authors:** Jan Stassen, Stephan M. Pio, See Hooi Ewe, Mohammed R. Amanullah, Kensuke Hirasawa, Steele C. Butcher, Gurpreet K. Singh, Kenny Y.K. Sin, Zee P. Ding, Nicholas W.S. Chew, Ching-Hui Sia, William K.F. Kong, Kian Keong Poh, David J. Cohen, Philippe Généreux, Martin B. Leon, Nina Ajmone Marsan, Victoria Delgado, Jeroen J. Bax

**Affiliations:** a Department of Cardiology, Leiden University Medical Center, Leiden, The Netherlands; b Department of Cardiology, National Heart Centre Singapore, Singapore, Singapore; c Department of Cardiology, Royal Perth Hospital, Perth, Western Australia, Australia; d Department of Cardiology, National University Heart Center Singapore, Singapore, Singapore; e Department of Cardiology, Saint Francis Hospital, Roslyn, New York, USA; f Cardiovascular Research Foundation, New York, New York, USA; g Gagnon Cardiovascular Institute, Morristown Medical Center, Morristown, New Jersey, USA; h Department of Cardiology, Columbia University Irving Medical Center/New York – Presbyterian Hospital, New York, New York, USA; i Turku Heart Center, University of Turku and Turku University Hospital, Turku, Finland

**Keywords:** All-cause mortality, Echocardiography, Left ventricular remodeling, Moderate aortic stenosis, Sex

## Abstract

**Background:**

Recent data showed poor long-term survival in patients with moderate AS. Although sex differences in left ventricular (LV) remodeling and outcome are well described in severe AS, it has not been evaluated in moderate AS.

**Methods:**

In this retrospective, multicenter study, patients with a first diagnosis of moderate AS diagnosed between 2001 and 2019 were identified. Clinical and echocardiographic parameters were recorded at baseline and compared between men and women. Patients were followed up for the primary endpoint of all-cause mortality with censoring at the time of aortic valve replacement.

**Results:**

A total of 1895 patients with moderate AS (age 73 ± 10 years, 52% male) were included. Women showed more concentric hypertrophy and had more pronounced LV diastolic dysfunction than men. During a median follow-up of 34 (13-60) months, 682 (36%) deaths occurred. Men showed significantly higher mortality rates at 3- and 5-year follow-up (30% and 48%, respectively) than women (26% and 39%, respectively) (*p* = 0.011). On multivariable analysis, male sex remained independently associated with mortality (hazard ratio 1.209; 95% CI: 1.024-1.428; *p* = 0.025). LV remodeling (according to LV mass index) was associated with worse outcomes (hazard ratio 1.003; CI: 1.001-1.005; *p* = 0.006), but no association was observed between the interaction of LV mass index and sex with outcomes.

**Conclusions:**

LV remodeling patterns are different between men and women having moderate AS. Male sex is associated with worse outcomes in patients with medically treated moderate AS. Further studies investigating the management of moderate AS in a sex-specific manner are needed.

## Introduction

Aortic stenosis (AS) is the most common valvular heart disease in developed countries^1^ with severe AS being associated with significantly reduced survival when left untreated.^2^^,^^3^ Recently, unfavorable clinical outcomes have also been reported in patients with moderate AS,^4^ challenging the optimal timing of valve intervention. The left ventricular (LV) remodeling response to pressure overload in severe AS seems different between sexes.^5^^,^^6^ In women, LV remodeling tends to be more concentric with a more preserved LV ejection fraction, whereas men more often show an eccentric hypertrophied LV with reduced LV systolic function.^7^^,^^8^ These sex differences may lead to different clinical and echocardiographic presentations, can influence assessment and management of AS, and may even affect patient survival, with recent studies showing less referral for aortic valve replacement (AVR) and excess mortality in women vs. men with severe AS.^9^^,^^10^ To better understand the different patterns of LV remodeling in men vs. women, looking at an earlier stage of the AS disease process may provide new insights, especially with recent studies demonstrating worse outcomes already in patients with moderate AS.^4^ However, sex-related differences in clinical presentation and outcomes in patients with moderate AS have not been evaluated. Therefore, the aim of the present study was to investigate the sex-related differences in clinical and echocardiographic presentation, as well as outcomes, of patients with medically treated moderate AS.

## Methods

### Patient Population

From the ongoing registries of patients with moderate aortic valve disease from 3 academic institutions (Leiden University Medical Center, Leiden, The Netherlands; National University Hospital, Singapore; and National Heart Center Singapore, Singapore), patients aged ≥18 years who presented between October 2001 and December 2019 (the registry of the National University Hospital, Singapore, started to include patients since 2011) with a first echocardiographic diagnosis of moderate AS were identified. Moderate AS was defined as an aortic valve area (AVA) between 1.0 and 1.5 cm^2^ with a dimensionless valve index between 0.25 and 0.50.^11^ Patients with previous aortic valve surgery, congenital heart disease, bicuspid aortic valve, supravalvular or subvalvular AS, or dynamic LV outflow tract obstruction were excluded. Of the 1895 patients included, 776 (41%) were included from the Leiden University Medical Center, 887 (47%) from the National Heart Center Singapore, and 232 (12%) from the National University Hospital Singapore. All patients underwent comprehensive clinical and echocardiographic evaluation at the time of first diagnosis of moderate AS. Patient information was prospectively collected in the departmental cardiology information system and retrospectively analyzed. Clinical data included demographic characteristics, cardiovascular risk factors, New York Heart Association (NYHA) functional class, and comorbidities. Data collection was consistent across the 3 centers. The study complies with the Declaration of Helsinki and was approved by the institutional review boards of each center. Due to the retrospective design of the study, the medical ethical committee of each participating center waived the need for written informed consent.

### Transthoracic Echocardiography

All echocardiographic studies were performed using commercially available ultrasound systems, and images were retrospectively analyzed by experienced echocardiographers in each center according to current guidelines.^12^ In the parasternal long-axis view, LV dimensions were assessed, and LV mass was calculated using Devereux’s formula and indexed for body surface area (left ventricular mass index [LVMi]).^12^ Relative wall thickness (RWT) was calculated with the following formula: (2 × posterior wall thickness)/LV internal diameter at end diastole.^12^ The echocardiographic variables that define LV geometry (LVMi and RWT) were subsequently used to categorize patients into 4 groups of cardiac remodeling: normal geometry (normal LVMi and RWT ≤0.42), concentric remodeling (normal LVMi and RWT >0.42), concentric hypertrophy (increased LVMi and RWT >0.42), and eccentric hypertrophy (increased LVMi and RWT ≤0.42).^12^ Normal LVMi was defined as LVMi ≤95 g/m^2^ for women and LVMi ≤115 g/m^2^ for men.^12^ LV volumes were assessed, and left ventricular ejection fraction (LVEF) was calculated according to the biplane Simpson’s method.^12^ Left atrial volumes were measured by the biplane method of disks and indexed for body surface area (left atrium volume index).^12^ From the apical 3- or 5-chamber views, continuous wave Doppler recordings were obtained to estimate peak aortic jet velocity.^13^ Mean and peak transvalvular pressure gradients were calculated using the Bernoulli equation.^13^ AVA was calculated using the LV outflow tract diameter and velocity time integrals of the aortic valve and LV outflow tract.^13^ Severity of mitral and tricuspid regurgitation was graded using a multiparametric approach, as recommended by current guidelines.^14^ Pulsed-wave Doppler recording of the transmitral flow was used to obtain peak early (E) and late (A) diastolic velocities.^15^ Using tissue Doppler imaging of the mitral annulus on the apical 4-chamber view, the e’ was measured at both the lateral and septal sides and averaged to calculate the E/e’ ratio.^15^ The right ventricular systolic pressure was calculated from the peak velocity of the tricuspid regurgitant jet according to the Bernoulli equation, adding the right atrial pressure determined by the inspiratory collapse and diameter of the inferior vena cava.^12^ For the evaluation of right ventricular systolic function, anatomical M-mode was applied on the focused apical 4-chamber view of the right ventricle to measure tricuspid annular plane systolic excursion.^12^

### Clinical Endpoints

Patients were followed up for the primary endpoint of all-cause mortality. Because current guidelines recommend a conservative approach for moderate AS,^11^^,^^16^ patients were censored for this analysis at the time of AVR or evolution to severe AS. The secondary endpoint was AVR at follow-up. Data on mortality were obtained from the departmental cardiology information system (EPD-Vision [The Netherlands] and Sunrise Clinical Manager System [Singapore]) linked to the governmental death registry database (i.e., the national registry controlled by the government that records information about the survival status of each inhabitant). Follow-up data were complete for all patients.

### Statistical Analysis

Continuous data are presented as mean ± standard deviation when normally distributed and as median (interquartile range) when not normally distributed. Categorical data are presented as frequencies and percentages. Continuous variables were compared using the independent sample Student t-test when normally distributed, whereas the Mann-Whitney U-test was used to compare continuous variables that did not adhere to a normal distribution. Categorical variables were compared using the Pearson chi-square test. Event-free survival curves were generated using the Kaplan-Meier method, and differences between groups were analyzed using the log-rank test. Patients were censored at the time of aortic valve intervention. Univariable and multivariable Cox proportional hazard analyses were performed to assess the association between sex and the endpoint of all-cause mortality. Clinical and echocardiographic variables were selected based on clinical importance. The following covariates, considered to have a potential prognostic impact, were included: age, arterial hypertension, diabetes mellitus, dyslipidemia, smoking, body mass index, coronary artery disease, previous myocardial infarction, atrial fibrillation, chronic obstructive pulmonary artery disease (COPD), stroke, estimated glomerular filtration rate, hemoglobin, NYHA functional class II-IV, LVEF, LVMi, AVA, moderate to severe mitral regurgitation, and moderate to severe tricuspid regurgitation. Variables that had a significant association on the univariable analysis (*p* < 0.05) were entered in the multivariable analysis. For both univariable and multivariable analyses, hazard ratios (HRs) and 95% confidence intervals (CIs) were calculated and reported. A 2-sided *p* value < 0.05 was considered statistically significant. A statistical analysis was performed using SPSS for Windows, version 25.0 (IBM, Armonk, New York).

## Results

### Clinical and Echocardiographic Characteristics

A total of 1895 patients (age 73 ± 10 years, 52% men) were included in the study. Baseline clinical characteristics are shown in Table 1, while Table 2 summarizes the echocardiographic data for the overall population. Most patients had arterial hypertension (80%) and dyslipidemia (75%), while diabetes mellitus was observed in more than one-third of the patients (35%). A history of coronary artery disease was present in 848 (45%) patients of whom 358 (19%) had a previous myocardial infarction. Dyspnea, defined as NYHA functional class ≥ II, was observed in 794 (42%) patients with 306 (16%) patients having severe symptoms (NYHA III-IV). The mean AVA was 1.21 ± 0.15 cm^2^, mean aortic mean pressure gradient 23 ± 8 mmHg, and mean peak aortic jet velocity 3.1 ± 0.6 m/s. The mean LVEF was 58 ± 13%.

**Table 1:** Clinical characteristics of the study population

Variable	Total population (n = 1895)	Female (n = 910)	Male (n = 985)	*p* value
Age, y	73.2 (±10.4)	73.5 (±10.8)	72.9 (±10.0)	0.277
Arterial hypertension (%)	1515 (80.2%)	733 (80.8%)	782 (79.6%)	0.491
Dyslipidemia (%)	1411 (74.7%)	675 (74.5%)	736 (74.9%)	0.824
Diabetes mellitus (%)	654 (34.6%)	332 (36.6%)	322 (32.8%)	0.079
Current smoker (%)	161 (8.9%)	41 (4.7%)	120 (12.7%)	<0.001
Obesity (%)	349 (18.9%)	196 (22.2%)	153 (15.8%)	<0.001
CAD (%)	848 (44.8%)	349 (38.4%)	499 (50.8%)	<0.001
Previous MI (%)	358 (18.9%)	138 (15.2%)	220 (22.4%)	<0.001
Atrial fibrillation (%)	555 (29.3%)	286 (31.5%)	269 (27.4%)	0.049
Previous stroke (%)	289 (15.3%)	126 (13.9%)	163 (16.6%)	0.102
COPD (%)	138 (7.3%)	36 (4.0%)	102 (10.4%)	<0.001
NYHA class II-IV (%)	794 (42.4%)	374 (41.6%)	420 (43.1%)	0.506
NYHA class III-IV (%)	306 (16.3%)	134 (14.9%)	172 (17.7%)	0.107
Angina (%)	167 (8.9%)	56 (6.2%)	111 (11.4%)	<0.001
Syncope (%)	31 (1.7%)	12 (1.3%)	19 (1.9%)	0.298
Beta-blocker (%)	938 (49.8%)	454 (50.3%)	484 (49.3%)	0.668
ACEi or ARB (%)	948 (50.3%)	426 (47.2%)	522 (53.2%)	0.009
MRA (%)	108 (5.8%)	48 (5.3%)	60 (6.1%)	0.463
Diuretic (%)	666 (35.3%)	321 (35.5%)	345 (35.1%)	0.850
CCB (%)	740 (39.3%)	387 (42.9%)	353 (35.9%)	0.002
Statin (%)	1320 (70.0%)	606 (67.1%)	714 (72.7%)	0.008
Aspirin (%)	884 (46.9%)	386 (42.7%)	498 (50.7%)	0.001
Oral anticoagulation (%)	388 (20.6%)	183 (20.3%)	205 (20.9%)	0.743
eGFR, mL/min/1.73 m^2^	67.2 (43.6-87.7)	65.1 (41.0-87.9)	69.1 (47.4-87.4)	0.256
Hemoglobin, g/dL	12.5 (11.0-13.7)	12.1 (10.7-13.1)	13.1 (11.3-14.4)	<0.001

*Notes*. Values are presented as mean ± SD, median (IQR) or n (%). Obesity is defined as a body mass index ≥30 kg/m^2^.

ACEi, angiotensin-converting enzyme inhibitor; ARB, angiotensin receptor blocker; CAD, coronary artery disease; CCB, calcium channel blocker; COPD, chronic obstructive pulmonary disease; eGFR, estimated glomerular filtration rate; IQR, interquartile range; MI, myocardial infarction; MRA, mineralocorticoid receptor antagonist; NYHA, New York Heart Association.

**Table 2:** Echocardiographic characteristics of the study population

Variable	Total population (n = 1895)	Female (n = 910)	Male (n = 985)	*p* Value
Left ventricle and atrium				
LV EDD, mm	47.9 (±7.4)	45.9 (±6.6)	49.8 (±7.6)	<0.001
LV ESV indexed, mL/m^2^	22 (17-31)	21 (16-28)	24 (18-35)	<0.001
LV EDV indexed, mL/m^2^	57 (46-73)	55 (44-71)	59 (48-73)	<0.001
LVEF, %	58 (±13)	60 (±11)	56 (±14)	<0.001
LVMI, g/m^2^	116.1 (±35.1)	110.6 (±32.4)	121.1 (±36.7)	<0.001
Stages of remodeling				<0.001
Normal geometry	326 (17.7%)	133 (15.2%)	193 (20.0%)	<0.05
Concentric remodeling	449 (24.4%)	175 (20.0%)	274 (28.3%)	<0.05
Concentric hypertrophy	658 (35.7%)	370 (42.3%)	288 (29.8%)	<0.05
Eccentric hypertrophy	409 (22.2%)	197 (22.5%)	212 (21.9%)	ns
LAVi, mL/m^2^	37 (29-47)	38 (30-50)	36 (29-46)	0.001
E/e’	14.5 (10.9-20.0)	15.6 (12.1-21.4)	13.4 (10.0-19.0)	<0.001
Moderate or severe MR (%)	185 (9.8%)	91 (10.0%)	94 (9.6%)	0.737
Aortic valve				
Stroke volume index, mL/m^2^	48 (±13)	51 (±14)	46 (±12)	<0.001
Peak aortic velocity, m/s	3.1 (±0.6)	3.0 (±0.6)	3.1 (±0.6)	0.012
Aortic mean pressure gradient, mmHg	23.2 (±8.4)	22.8 (±7.9)	23.5 (±8.7)	0.073
Aortic valve area, cm^2^	1.21 (±0.15)	1.20 (±0.15)	1.23 (±0.15)	<0.001
Aortic valve area indexed, cm^2^/m^2^	0.72 (±0.12)	0.76 (±0.13)	0.68 (±0.11)	<0.001
DVI	0.34 (±0.06)	0.35 (±0.06)	0.33 (±0.06)	<0.001
Moderate or severe AR, %	213 (11.2%)	108 (11.9%)	105 (10.7%)	0.401
Right ventricle				
TAPSE, mm	21 (19-24)	21 (19-24)	21 (18-25)	0.273
PASP, mmHg	33 (27-41)	34 (28-42)	33 (26-41)	0.001
Moderate or severe TR, %	301 (16.0%)	173 (19.1%)	128 (13.1%)	<0.001

*Notes*. Values are presented as mean ± SD, median (IQR) or n (%).

AR, aortic regurgitation; DVI, dimensionless valve index; EDD, end-diastolic diameter; EDV, end-diastolic volume; ESV, end-systolic volume; LAVi, left atrial volume index; LV, left ventricular; LVEF, left ventricular ejection fraction; LVMI, left ventricular mass index; MR, mitral regurgitation; PASP, pulmonary artery systolic pressure; TAPSE, tricuspid annular plane systolic excursion; TR, tricuspid regurgitation.

**Figure 1:**
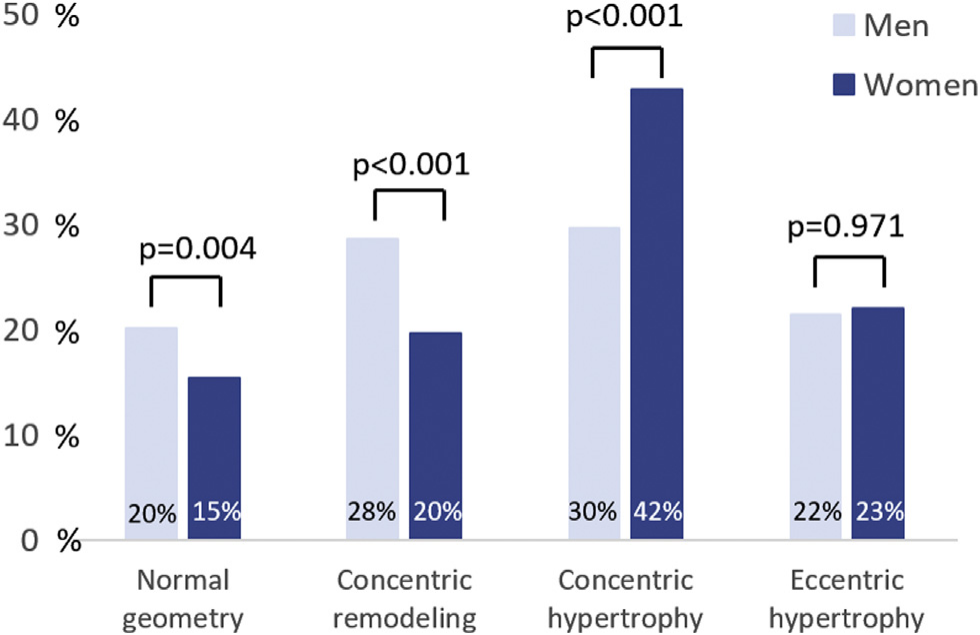
**Left ventricular remodeling in patients with moderate aortic stenosis according to sex.** Bar chart demonstrating the frequency of the left ventricular remodeling patterns and the distribution according to sex.

There was no difference in age between men and women at first presentation of moderate AS. However, women were more often obese than men (22% vs. 16%, *p* < 0.001), while men were more likely to have coronary artery disease (51% vs. 38%, *p* < 0.001), previous myocardial infarction (22% vs. 15%; *p* < 0.001), and COPD (10% vs. 4%; *p* < 0.001). Although men had more angina at baseline (11% vs. 6%; *p* < 0.001), there was no difference in the percentage of men and women having dyspnea NYHA class ≥ II (43% vs. 42%; *p* = 0.506). In terms of echocardiographic data, men had larger indexed LV end-diastolic and end-systolic volumes than women (59 [48-73] vs. 55 [44-71] mL/m^2^ and 24 [18-35] vs. 21 [16-28] mL/m^2^, respectively; *p* < 0.001 for both), and LVEF was lower in men than in women (56 ± 14% vs. 61 ± 11%; *p* < 0.001). In addition, LVMi was significantly lower in female patients than that in male patients (111 ± 32 vs. 121 ± 37 g/m^2^; *p* < 0.001). Yet, the prevalence of concentric hypertrophy was higher in women (42% vs. 30%; *p* < 0.001) (Figure 1), and LV diastolic dysfunction was more pronounced with higher E/e’ (16 [12-21] vs. 13 [10-19]; *p* < 0.001), larger left atrium volume index (38 [30-50] vs. 36 [29-46] mL/m^2^; *p* = 0.001), and higher systolic pulmonary artery pressures (34 [28-42] vs. 33 [26-41] mmHg; *p* = 0.001). Men showed a slightly higher peak aortic jet velocity (3.1 ± 0.6 vs. 3.0 ± 0.6 m/s; *p* = 0.012) and had a lower indexed aortic valve area (0.68 ± 0.11 vs. 0.76 ± 0.13 cm^2^/m^2^; *p* < 0.001).

### Association Between Sex and Survival

After a median follow-up of 34 (13-60) months, 605 (32%) patients were censored because of AVR or diagnosis of severe AS. Of these 605 patients, 178 (9%) patients underwent AVR because of moderate AS with concomitant CABG (as recommended by the current guidelines),^11^^,^^16^ and 427 (23%) patients progressed to severe AS, all of whom subsequently underwent AVR. Women (n = 249) were less likely to undergo AVR than men (n = 356) (27% vs. 36%; *p* < 0.001). The mean time from diagnosis of moderate AS to intervention was 19 (6 – 39) months for men and 29 (12 – 52) months for women (*p* < 0.001).

**Figure 2:**
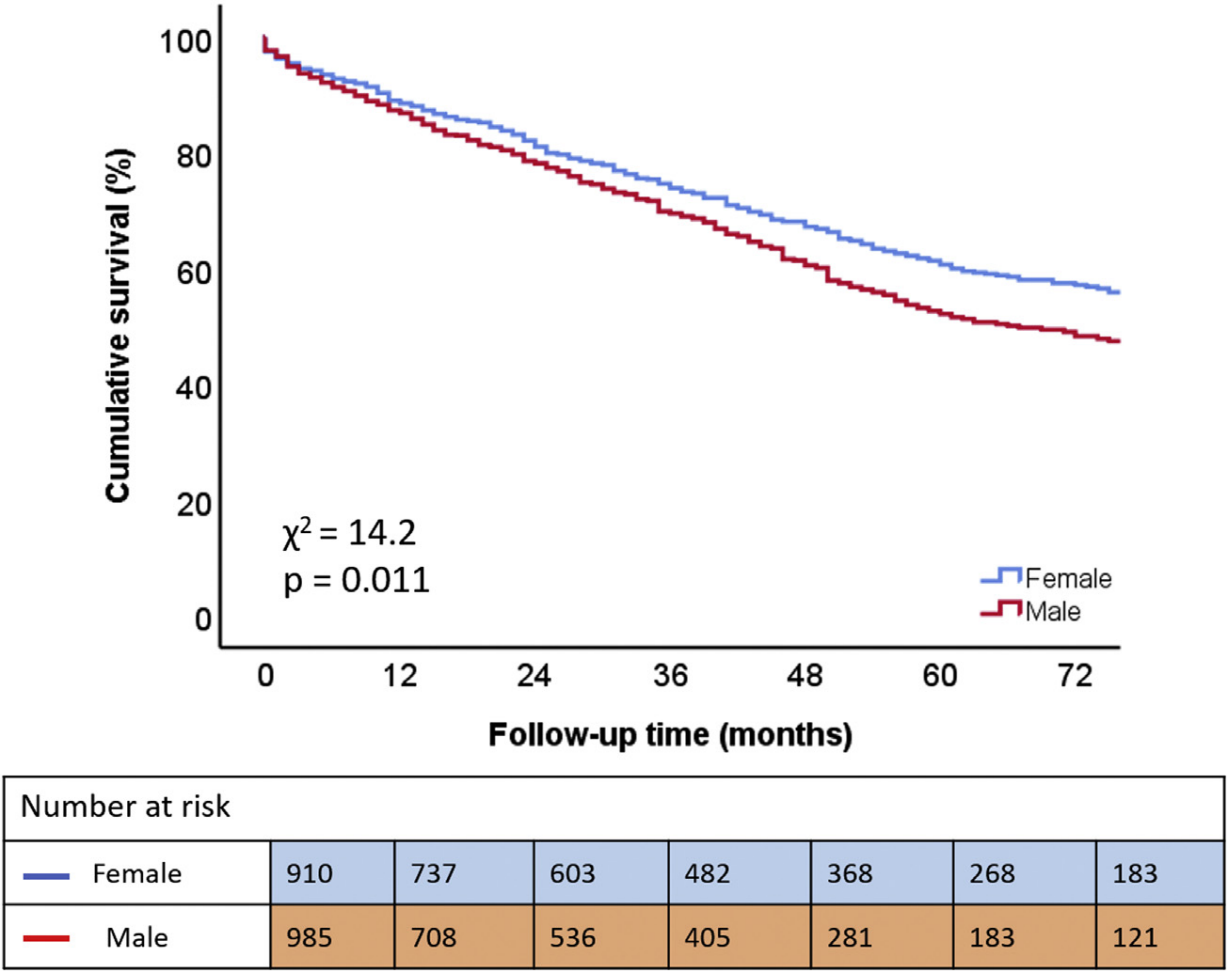
**Kaplan-Meier curve for time to cumulative survival, according to sex**.

During the follow-up period of 34 (13-60) months, 682 (36%) deaths occurred. The Kaplan-Meier survival curves demonstrated significantly lower survival rates at 3- and 5-year follow-up for men (70% and 52%, respectively) than for women (74% and 61%, respectively) (*p* = 0.025; Figure 2). Moreover, multivariable Cox regression analysis, adjusting for relevant covariates (i.e., age, arterial hypertension, diabetes mellitus, dyslipidemia, body mass index, smoking, coronary artery disease, previous myocardial infarction, atrial fibrillation, COPD, stroke, estimated glomerular filtration rate, hemoglobin, NYHA functional class II-IV, LVEF, LVMi, AVA, moderate to severe MR, and moderate to severe TR) showed that male sex remained independently associated with worse outcomes (HR 1.209; 95% CI: 1.024-1.428; *p* = 0.025; Table 3). Male sex also remained independently associated with the secondary outcome (i.e., AVR at follow-up) on the univariable analysis (HR 1.272; 95% CI: 1.081-1.497; *p* = 0.004) as well as on the multivariable analysis (HR 1.230; 95% CI: 1.024-1.478; *p* = 0.027) (adjusting for the same variables that were used in the multivariable Cox regression analysis for the primary endpoint).

**Table 3:** Univariable and multivariable Cox regression analysis to assess the association between sex and all-cause mortality

Variable	Univariable analysis		Multivariable analysis	
	HR (95% CI)	*p* value	HR (95% CI)	*p* value
Male sex	1.214 (1.045-1.412)	0.007	1.209 (1.024-1.428)	0.025
Age, y	1.036 (1.028-1.044)	<0.001	1.031 (1.022-1.040)	<0.001
Arterial hypertension	1.392 (1.133-1.710)	0.002	0.904 (0.721-1.132)	0.379
Diabetes mellitus	1.619 (1.390-1.886)	<0.001	1.485 (1.255-1.756)	<0.001
Dyslipidemia	0.998 (0.839-1.187)	0.982		
BMI, kg/m^2^	0.961 (0.946-0.977)	<0.001	0.967 (0.950-0.984)	<0.001
Current smoker	1.134 (0.873-1.473)	0.345		
Coronary artery disease	1.198 (1.030-1.394)	0.019	0.912 (0.767-1.083)	0.293
Previous myocardial infarction	1.481 (1.237-1.772)	<0.001	0.977 (0.793-1.203)	0.827
Atrial fibrillation	1.261 (1.075-1.479)	0.004	1.141 (0.957-1.361)	0.141
COPD	1.113 (0.820-1.512)	0.492		
Stroke	1.419 (1.173-1.717)	<0.001	1.187 (0.964-1.462)	0.107
NYHA class II-IV	2.332 (2.003-2.716)	<0.001	1.803 (1.528-2.127)	<0.001
Estimated eGFR, mL/min/1.73 m^2^	0.984 (0.981-0.986)	<0.001	0.985 (0.982-0.988)	<0.001
Hemoglobin, g/dL	0.989 (0.840-1.185)	0.115		
LVEF, %	0.976 (0.970-0.981)	<0.001	0.987 (0.981-0.994)	<0.001
LVMi, g/m^2^	1.007 (1.005-1.009)	<0.001	1.003 (1.001-1.005)	0.006
AVA, cm^2^	0.564 (0.339-0.939)	0.028	1.264 (0.728-2.194)	0.404
Moderate to severe MR	1.543 (1.223-1.946)	<0.001	0.975 (0.749-1.269)	0.850
Moderate to severe TR	1.713 (1.426-2.058)	<0.001	1.488 (1.212-1.828)	<0.001

AVA, aortic valve area; BMI, body mass index; COPD, chronic obstructive pulmonary disease; eGFR, estimated glomerular filtration rate; HR, hazard ratio; LVEF, left ventricular ejection fraction; LVMi, left ventricular mass index; MR, mitral regurgitation; NYHA, New York Heart Association; TR, tricuspid regurgitation.

### Association Between Sex Differences in LV Remodeling and Survival

**Figure 3:**
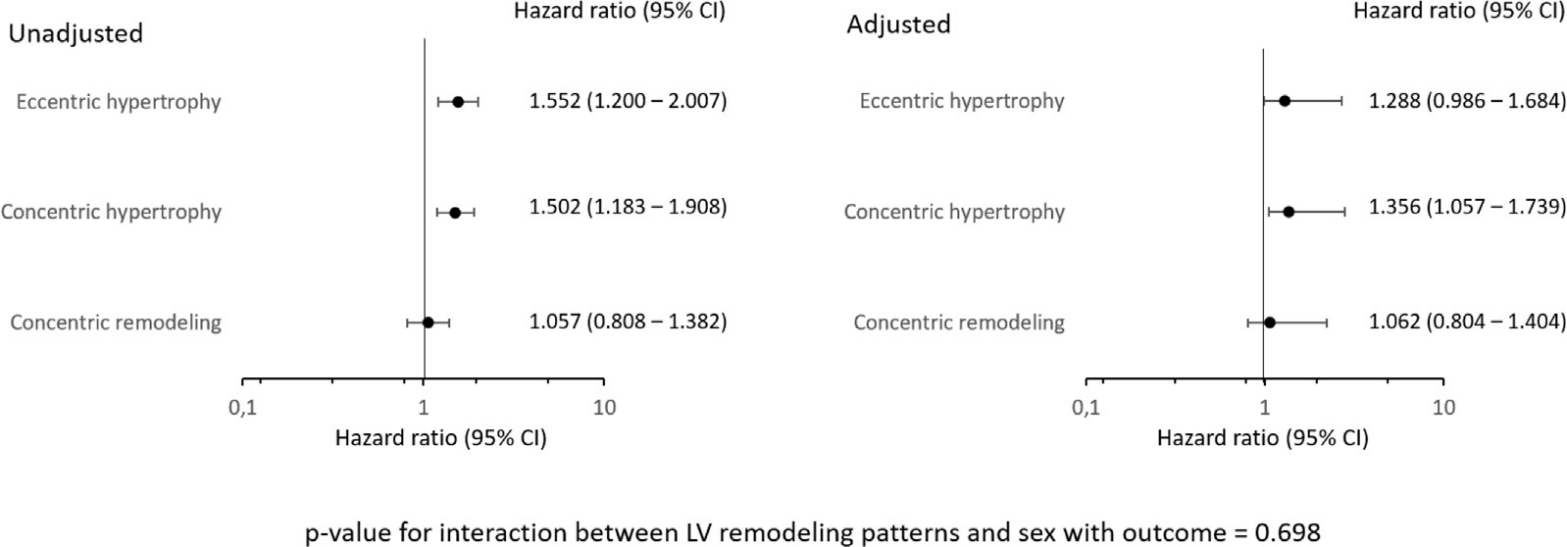
**Forest plots of unadjusted and adjusted hazard ratios for long-term mortality according to different LV remodeling patterns (using normal geometry as the reference group)**. Abbreviation: LV, left ventricular.

On multivariable Cox regression analysis, more pronounced LV remodeling (defined as higher LVMi) was associated with worse outcomes (HR 1.003; 95% CI: 1.001-1.005; *p* = 0.006; Table 3). However, there was no significant association of LVMi and sex, as an interaction term, with outcome (*p* = 0.097). When dividing the study population according to different LV remodeling patterns, concentric hypertrophy (HR 1.502; 95% CI: 1.183-1.908; *p* = 0.001; Table 4) and eccentric hypertrophy (HR 1.552; 95% CI: 1.200-2.007; *p* = 0.001; Table 4) were significantly associated with worse outcome on the univariable analysis. However, on the multivariable Cox regression analysis, only concentric hypertrophy remained independently associated with outcome (HR 1.356; 95% CI: 1.057-1.739; *p* = 0.016; Table 4). There was no significant interaction between LV remodeling patterns and sex with outcome (*p* = 0.698). Figure 3 shows the forest plots of the unadjusted and adjusted HRs for long-term mortality according to the different LV remodeling patterns.

**Table 4:** Univariable and multivariable Cox regression analysis to assess the association between different LV remodeling patterns and all-cause mortality

Variable	Univariable analysis		Multivariable analysis	
	HR (95% CI)	*p* value	HR (95% CI)	*p* value
Male sex	1.214 (1.045-1.412)	0.007	1.253 (1.058-1.484)	0.009
Age, y	1.036 (1.028-1.044)	<0.001	1.034 (1.026-1.043)	<0.001
Arterial hypertension	1.392 (1.133-1.710)	0.002	0.871 (0.695-1.091)	0.230
Diabetes mellitus	1.619 (1.390-1.886)	<0.001	1.525 (1.291-1.802)	<0.001
Dyslipidemia	0.998 (0.839-1.187)	0.982		
BMI, kg/m^2^	0.961 (0.946-0.977)	<0.001	0.849 (0.674-1.069)	0.164
Current smoker	1.134 (0.873-1.473)	0.345		
Coronary artery disease	1.198 (1.030-1.394)	0.019	0.922 (0.775-1.096)	0.356
Previous myocardial infarction	1.481 (1.237-1.772)	<0.001	0.967 (0.784-1.193)	0.752
Atrial fibrillation	1.261 (1.075-1.479)	0.004	1.184 (0.995-1.410)	0.057
COPD	1.113 (0.820-1.512)	0.492		
Stroke	1.419 (1.173-1.717)	<0.001	1.191 (0.968-1.467)	0.099
NYHA class II-IV	2.332 (2.003-2.716)	<0.001	1.803 (1.528-2.127)	<0.001
Estimated eGFR, mL/min/1.73 m^2^	0.984 (0.981-0.986)	<0.001	0.984 (0.982-0.987)	<0.001
Hemoglobin, g/dL	0.989 (0.840-1.185)	0.115		
LVEF, %	0.976 (0.970-0.981)	<0.001	0.982 (0.976-0.988)	<0.001
AVA, cm^2^	0.564 (0.339-0.939)	0.028	1.146 (0.663-1.983)	0.625
Moderate to severe MR	1.543 (1.223-1.946)	<0.001	1.043 (0.800-1.359)	0.757
Moderate to severe TR	1.713 (1.426-2.058)	<0.001	1.580 (1.288-1.937)	<0.001
Normal geometry	Reference		Reference	
Concentric remodeling	1.057 (0.808-1.382)	0.687	1.062 (0.804-1.404)	0.672
Concentric hypertrophy	1.502 (1.183-1.908)	0.001	1.356 (1.057-1.739)	0.016
Eccentric hypertrophy	1.552 (1.200-2.007)	0.001	1.288 (0.986-1.684)	0.064

AVA, aortic valve area; BMI, body mass index; COPD, chronic obstructive pulmonary disease; eGFR, estimated glomerular filtration rate; HR, hazard ratio; LV, left ventricular; LVEF, left ventricular ejection fraction; MR, mitral regurgitation; NYHA, New York Heart Association; TR, tricuspid regurgitation.

## Discussion

The main findings of this study with data obtained from a large registry of patients with moderate AS can be summarized as follows: 1) Sex-related differences in LV remodeling are apparent in moderate AS, with women showing more LV concentric hypertrophy and more pronounced LV diastolic dysfunction than men, and 2) male sex is independently associated with worse outcomes in medically treated moderate AS after adjusting for various clinical and echocardiographic variables.

### Sex-Related Differences in LV Remodeling in Moderate AS

AS is characterized by progressive narrowing of the aortic valve, causing LV pressure overload. The LV responds to this pressure overload by triggering a hypertrophic response, leading to an increase in myocyte size and LV mass. It has been suggested that the pathophysiology of AS at the ventricular level differs between men and women. For a similar AS severity, studies have shown that women develop more concentric hypertrophy with a smaller LV cavity size, whereas men more often present with eccentric hypertrophy and depressed LV systolic function.^17^, ^18^, ^19^ However, most of these studies have been focusing on patients with severe AS, and studies specifically addressing sex-related differences in moderate AS are not available. In this regard, our data expand on previous studies by demonstrating that sex-related differences in LV remodeling are already present at an earlier stage of the AS disease process.

The susceptibility to LV remodeling and fibrosis in men vs. women has been partially attributed to sex hormones, such as estrogen.^20^^,^^21^ Furthermore, activation of the renin-angiotensin-aldosterone system is believed to play a role in the LV remodeling process as well, with some evidence suggesting that this process may predominantly affect female patients.^22^ Other mechanisms that could play a role are sex-specific expression of matrix-related genes and differences in the regulation of collagen synthesis.^23^ Further studies seem necessary to determine the underlying mechanisms for the sex differences seen in LV remodeling in moderate AS.

Although the differences in LV remodeling may reflect the underlying differences in cardiac comorbidities between men and women, there was no difference in age, concomitant arterial hypertension, dyslipidemia, diabetes mellitus, or chronic kidney disease that could fully explain the higher prevalence of concentric hypertrophy in women vs. men. Because AS severity is a continuous process with each incremental increase in AS severity imposing an increased pressure load on the LV, it is not unlikely that moderate AS itself provides an important contribution to the LV remodeling process as well. Conversely, the impact of the underlying comorbidities on LV remodeling in moderate AS might explain why some patients with severe AS do not show complete reverse remodeling after AVR.

### Sex-Related Differences in Outcomes in Moderate AS

Although LV remodeling is initially considered beneficial, maintaining normal wall stress and systolic function, it eventually becomes maladaptive, leading to a myocardial oxygen supply-demand mismatch with repetitive ischemia which results in myocardial fibrosis.^24^^,^^25^ It is well known that myocardial fibrosis eventually leads to a progressive impairment in LV diastolic relaxation and LV performance and is therefore associated with increased morbidity and mortality.^26^, ^27^, ^28^ Previous studies have shown that women have worse outcomes than men in severe AS,^9^^,^^10^ but recent studies including patients with moderate AS have shown different results. Strange et al.^4^ demonstrated poor survival rates in patients with moderate AS, with male sex being independently associated with worse outcomes. In another study, including 305 patients with moderate AS and LV systolic dysfunction, van Gils et al.^29^ showed that male sex was independently associated with the combined endpoint of death, AVR, or hospitalization. Our findings, demonstrating that male sex is independently associated with worse outcomes in patients with medically treated moderate AS, support these observations.

Heart failure is the main cardiac cause of death in patients having severe AS.^30^ In the current study, almost half of the study population (44%) already presented with symptoms of heart failure, defined as NYHA functional class II or more, and all-cause mortality was remarkably high (36%). Interestingly, women more often showed a phenotype of “heart failure with preserved ejection fraction,” having more concentric hypertrophy, more preserved LVEF, and more pronounced LV diastolic dysfunction. In contrast, men more often presented with a phenotype of “heart failure with reduced ejection fraction,” having more coronary artery disease, larger LV dimensions, lower LVEF, and less concentric hypertrophy. Although comorbidities could partially explain the different phenotypes of heart failure and therefore the higher mortality rates observed in men vs. women, male sex remained independently associated with worse outcomes after adjusting for other factors known to have an impact on long-term prognosis (e.g., age, arterial hypertension, diabetes mellitus, dyslipidemia, body mass index, coronary artery disease, previous myocardial infarction, atrial fibrillation, COPD, estimated glomerular filtration rate, NYHA functional class, LVEF, and LVMi). Other mechanisms explaining the sex-related discrepancy on outcomes in medically treated moderate AS could therefore also play a role. In a study of patients with severe AS without coronary artery disease, Carroll et al.^5^ reported men have the tendency to develop significantly higher LV wall stress than women, despite having the same degree of LV outflow obstruction. Similarly, animal research using a spontaneous hypertensive rat model showed that male rats develop cardiac failure earlier than female rats with a significant decrease in LV performance in age-matched male vs. female rats.^31^ Furthermore, Petrov et al.^32^ demonstrated men to have more fibrosis-associated genes and more fibrosis than women at the time of isolated AVR despite having the same degree of AS stenosis. These findings may suggest that male patients are less able to cope with the same degree of afterload than female patients, contributing to the observation that male patients with moderate AS have worse prognosis. The current study also shows that men may experience more rapid disease progression than women, perhaps contributing to the worse prognosis. However, further studies are necessary to confirm these findings.

### Clinical Implications

Current guidelines recommend a “watchful waiting” approach for patients with moderate AS.^11^^,^^16^ However, contemporary evidence suggests that moderate AS is not as benign as commonly assumed, especially in the presence of concurrent LV systolic dysfunction.^29^^,^^33^ Therefore, the Transcatheter Aortic Valve Replacement to UNload the Left ventricle in patients with ADvanced heart failure (TAVR UNLOAD) trial (NCT02661451) is currently exploring the hypothesis that transcatheter AVR could improve outcomes in these patients.^34^ Because male patients not only more often present with LV systolic dysfunction but also seem less able to cope with the same increase in afterload compared to women,^5^^,^^31^^,^^32^ questions of optimizing the management of AS in a sex-specific manner are rising. However, whether the timely implementation of AVR will translate into improved outcomes will require prospective evaluation.

### Limitations

This study is subject to the limitations of a retrospective, observational design. A time span of 18 years was used to retrospectively identify patients to acquire the large cohort as presented although the length of the inclusion period was different between the centers. Echocardiographic measurements of LV myocardial mass are less accurate than magnetic resonance imaging or computed tomography, but they are the most widely available and most often used to assess LV hypertrophy in patients with cardiovascular diseases. In addition, other determinants besides AS severity could be considered to affect the magnitude and pattern of LV remodeling. All-cause mortality was chosen as the primary endpoint since the exact cause of death could not be determined in all patients.

## Conclusion

In this large series of patients with medically treated moderate AS, LV remodeling patterns were different between men and women, and men had worse survival rates than women. Further studies investigating the management of moderate AS in a sex-specific manner are needed.

## Ethics Statement

The present research has adhered to the relevant ethical guidelines. Informed consent was waived by the Institutional Review Board due to the retrospective nature of the study.

## Funding

Jan Stassen received funding from the 10.13039/501100000860European Society of Cardiology (ESC Training Grant App000064741). Stephan M. Pio received funding from the 10.13039/501100000860European Society of Cardiology (ESC Training Grant T-2018-17405). Steele C. Butcher received funding from the 10.13039/501100000860European Society of Cardiology (ESC Research Grant App000080404).

## Disclosures statement

The Department of Cardiology, Heart Lung Center, Leiden University Medical Centre, received research grants from 10.13039/100011949Abbott Vascular, 10.13039/100004326Bayer, 10.13039/501100005035Biotronik, Bioventrix, 10.13039/100008497Boston Scientific, 10.13039/100006520Edwards Lifesciences, 10.13039/100006775GE Healthcare, Ionis, and 10.13039/100004374Medtronic. Jeroen J. Bax received speaker fees from Abbott Vascular. Nina Ajmone Marsan received speaker fees from Abbott Vascular and GE Healthcare. Victoria Delgado received speaker fees from Abbott Vascular, Edwards Lifesciences, GE Healthcare, Medtronic, MSD, and Novartis. The remaining authors have nothing to disclose.
